# Navigating adulthood with juvenile-onset type 1 diabetes: a scoping review on education, employment and quality of life

**DOI:** 10.3389/fpubh.2025.1707581

**Published:** 2025-12-17

**Authors:** Eleonora Maurel, Arianna Fornari, Alessandra Knowles, Erika Guastafierro, Martina Lanza, Alessia Marcassoli, Matilde Leonardi, Luca Ronfani, Lorenzo Monasta

**Affiliations:** 1 Clinical Epidemiology and Public Health Research Unit, Institute for Maternal and Child Health-IRCCS Burlo Garofolo, Trieste, Italy; 2 Neurology, Public Health and Disability Unit, Fondazione IRCCS Istituto Neurologico Carlo Besta, Milano, Italy

**Keywords:** type 1 diabetes, educational achievement, employment, health-related quality of life, mental health

## Abstract

**Background:**

Juvenile type 1 diabetes can present lifelong challenges that may affect educational, employment, and health-related quality of life (HRQoL) outcomes in adulthood. This scoping review aims at exploring the long-term impact of juvenile-onset type 1 diabetes on education and career achievements and well-being in adulthood.

**Methods:**

A scoping review conducted using PubMed and PsycInfo (2005–2025) identified studies comparing adults diagnosed with type 1 diabetes during childhood and adolescence to controls without type 1 diabetes. Three studies focusing on educational attainment, employment, income, and HRQoL, met the inclusion criteria.

**Results:**

The studies included in the review show that university attendance is significantly lower among individuals with juvenile-onset type 1 diabetes, and this affects both men and women. Access to the labor market and earnings are similarly affected, especially in the case of women who experience both lower employment rates and income, compared to controls without diabetes. Men with type 1 diabetes have equal opportunities for workforce entry, but this does not translate into long-term income parity. Compared to healthy controls, HRQoL is lower among adults with type 1 diabetes, particularly in the school/work and emotional domains. Common challenges include low energy, forgetfulness, and concentration difficulties.

**Conclusion:**

While it is a matter of debate how juvenile-onset type 1 diabetes affects academic achievements, there is general agreement that it leads to persistent disadvantages in employment and HRQoL in adulthood. Targeted support from diagnosis and during transition to adulthood is essential to mitigate the long-term impact of juvenile-onset type 1 diabetes on educational, employment and psychosocial outcomes for this population.

## 1 Background

Type 1 diabetes (T1D) is a chronic condition often diagnosed in childhood or adolescence, requiring lifelong management and regular monitoring to prevent serious health complications (1, 2). For individuals with juvenile-onset type 1 diabetes, the transition from pediatric to adult care introduces unique challenges. This shift entails not only a change in healthcare providers and settings but also increased expectations of self-management, occurring precisely during a period marked by major life changes (3, 4).

As these individuals move into adulthood, they frequently encounter additional challenges in various areas of life. Studies suggest that young adults with juvenile-onset type 1 diabetes face difficulties in formal education due to health-related absenteeism, concentration issues, and the demands of self-management in academic settings (5, 6). Furthermore, the influence of a chronic illness on psychosocial development can affect family relationships and peer interactions, creating potential stressors that carry into adult life (7). Similarly, employment and salary prospects may be negatively impacted by the effects of T1D, both directly, in terms of time lost for medical care and self-management, and indirectlyby the mental health burden associated with chronic illnesses (8, 9).

Despite these known challenges, there is a significant gap in the literature regarding the long-term impacts of juvenile-onset type 1 diabetes on adult life. In particular, the interplay between self-management and outcomes such as academic achievement, career advancement, salary, employment stability, and quality of life (QoL), including mental and physical health, is not fully understood (10–12). This study aims at exploring these outcomes and identifying potential barriers that adults diagnosed with type 1 diabetes during childhood or adolescence may face. Through in-depth analysis of these factors, this research seeks to provide insights that could inform more comprehensive support systems during the transition to adulthood, ultimately improving QoL and long-term outcomes for individuals with type 1 diabetes.

This scoping review is part of the activities of Work Package 11 of the Joint Action on CARdiovascular diseases and Diabetes (JACARDI), whose objective is to reduce the burden of cardiovascular disease and diabetes across Europe. Work Package 11 focuses specifically on identifying barriers and implementing solutions to support continued or renewed labor market participation for people living with cardiovascular disease or diabetes, contributing to their overall well-being and economic inclusion.

## 2 Methods

We conducted a scoping review to examine the long-term impact of juvenile-onset type 1 diabetes on adult achievements and QoL. This methodology was deemed appropriate because of the varied nature of the research exploring this topic, as well as the heterogeneity of the methodologies used to evaluate it. We aimed to identify literature gaps on the topic of barriers that create inequalities between adults with juvenile-onset T1D and controls without T1D. Therefore, we established to limit our review to quantitative studies with a case–control design and deemed it sufficient to consider one multidisciplinary (PubMed/MEDLINE) and one subject-specific database (PsychINFO). We are aware that qualitative studies and gray literature offer important insights, however, in order for comparisons to be drawn, gaps and barriers need to be measured against a counterfactual, leaving the qualitative aspects aside. We limited our search to articles published from 2005 to 2025 because knowledge and technology have changed radically in the last 20 years, producing a significant impact on the quality of life of people living with T1D.

This review was conducted following the Preferred Reporting Items for Systematic Reviews and Meta-Analyses extension for Scoping Reviews (PRISMA-ScR) guidelines (13).

### 2.1 Search strategy

On January 22nd, 2025, a search was conducted on PUBMED and PsycInfo. Results were uploaded to the Rayyan platform for selection (14).

The search strings were:

- For Pubmed: ((adult[MeSH Terms]) OR (adult, young[MeSH Terms])) AND ((type 1 diabetes mellitus[MeSH Terms]) OR (diabetes mellitus, type 1[MeSH Terms])) AND ((physical activity[MeSH Terms]) OR (employment[MeSH Terms]) OR (marital status[MeSH Terms]) OR (educational status[MeSH Terms]) OR (achievement, educational[MeSH Terms]) or (factor, psychosocial[MeSH Terms]) OR (factors, psychosocial[MeSH Terms])) AND (consequence* OR impact* OR gap OR gaps).
- For PsycInfo: tiab(adult) AND tiab(type 1 diabetes mellitus) AND tiab(“psychosocial distress” OR “education” OR “schooling” OR “quality of life” OR “mental health” OR “distress” OR “depression” OR “anxiety” OR “satisfaction” OR “income” OR “salary” OR “job” OR “employment”).

### 2.2 Eligibility criteria

Studies were included in the review if they met the following criteria:

- Publication date: From 2005 to 2025;
- Population: The studies must involve adults aged ≥18 years;
- Diagnosis: Participants must have been diagnosed with T1D during childhood or adolescence;
- Control group: The studies must include a control group consisting of individuals who did not receive a diagnosis of T1D during childhood or adolescence;
- Gaps and dimensions: The studies should examine any gaps or differences between the T1D group and the control group regarding dimensions such as formal education, employment, salaries, physical activity, social/family interactions, and QoL.

Studies were excluded if they had the following characteristics:

- Study type: Reviews and gray literature documents were excluded;
- Language: Articles not published in English were excluded;
- Research Method: Qualitative studies were excluded.

### 2.3 Study selection

The titles and abstracts were screened by two reviewers (EM and EG) independently. The full text of the selected articles was then read and assessed against eligibility criteria. Any disagreements were subsequently resolved through discussion between reviewers until consensus was achieved.

**Figure 1:**
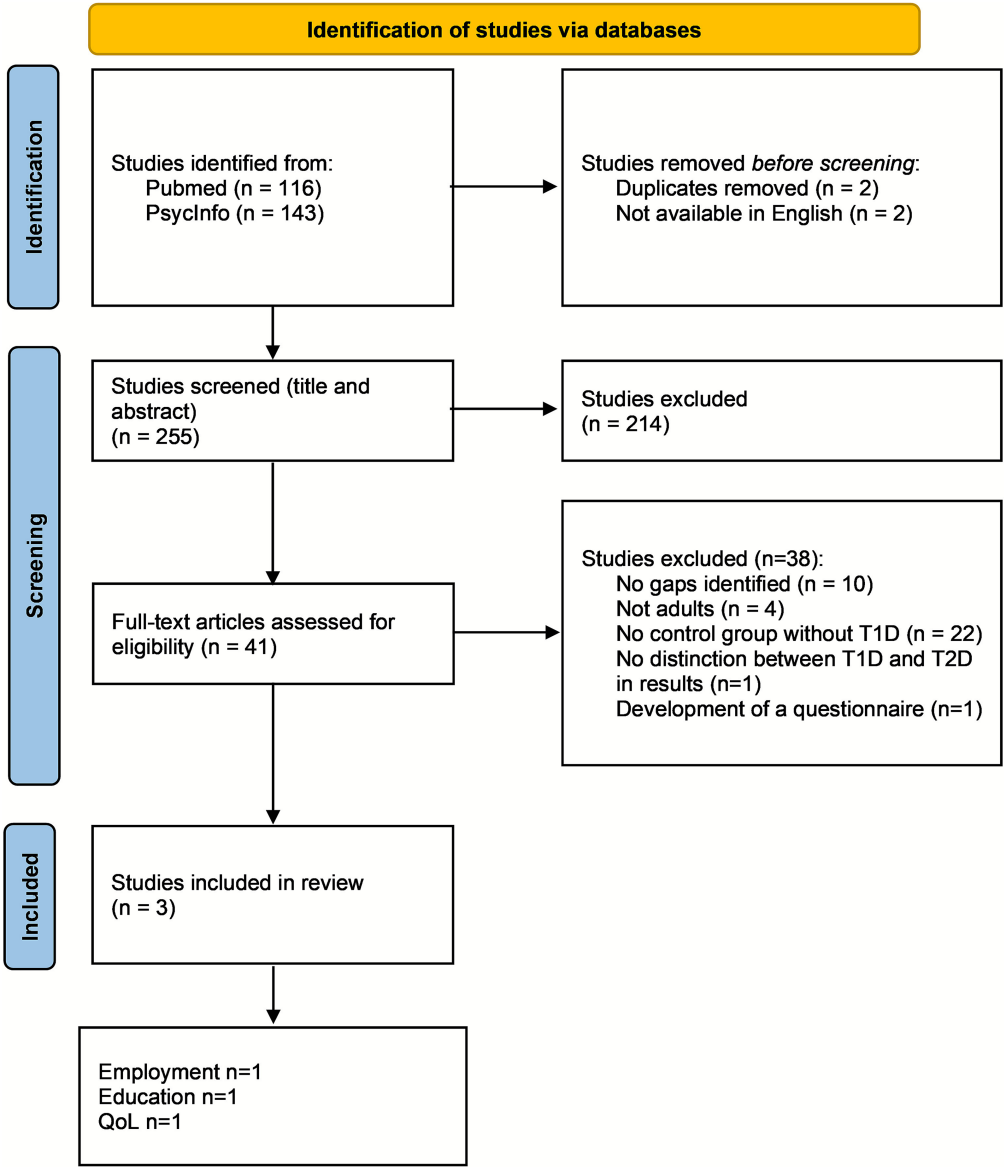
PRISMA flow chart.

A summary of the PRISMA flow chart can be found in Figure 1.

Data were extracted into an Excel spreadsheet, developed specifically for this review, to classify the studies according to themes and organize the relevant information (e.g., dimension of the gap, population characteristics, onset of disease, type of experimental design, presence of a control group without type 1 diabetes).

## 3 Results

### 3.1 Search results and characteristics of studies

The search strategy designed for the present scoping review identified 259 articles potentially investigating the gaps and barriers that adults diagnosed with T1D during childhood or adolescence may face: 116 from PubMed and 143 from PsycInfo. Out of the total 259 articles, two duplicates and two that were not available in English were removed.

Following title/abstract screening of the remaining 255 articles, 41 abstracts were retained. The result of the full-text screening of the 41 articles was that 10 did not identify any gap, 4 did not include the adult population, 22 did not compare results between individuals with T1D and controls without T1D, 1 did not make a distinction between T1D and Type 2 Diabetes in the results, and 1 had the sole objective of developing a questionnaire. The 41 articles that underwent full-text screening are described in Supplementary Table S1. Three articles met the eligibility criteria and were therefore included in the final review (15–17). Of these, one mainly focused on formal education gaps (15), one on employment gaps (16), and one on health-related quality of life (HRQoL) (17). No articles fitting the criteria were found that presented results on physical activity and/or marital status outcomes in individuals with T1D. The articles included in the analysis are briefly described in Table 1.

**Table 1:** Description of the included articles.

Study	Study type	Description of population	Sample population with T1D (*n*)	Control population	Questionnaire(s), Test(s), other instruments / Data source for identifying the gap	Dimensions
Bronne et al. (17)	Cross sectional study	Young Adults with T1D (mean age 22.7 ± 1.6 years)	165	310 healthy young adults; 75 young adults with a chronic disease	PedsQL-YA	Physical health; Emotional functioning; Social functioning; School/Work functioning
Lovén et al. (15)	Retrospective observational cohort study	Adults with T1D (born 1962–1975; age at diagnosis: 2 to 15 years)	2,756	11,020 adults without T1D from the Swedish Total Population Register	Swedish Childhood Diabetes Register, Multi-Generation Register, Longitudinal Integration Database for Health Insurance and Labor Market Studies, Swedish Register of Education	Level and field of upper secondary education; University attendance; Health oriented university programs
Persson et al. (16)	Retrospective observational cohort study	Adults with T1D (born 1972–1978; age at diagnosis: <15 years)	2,485	9,940 adults without T1D from the Swedish Total Population Register	Swedish Childhood Diabetes Register, Multi-Generation Register, Longitudinal Integration Database for Health Insurance and Labor Market Studies, Swedish Medical Birth Register	Employment; Earnings; Educational level; Likelihood of having children

Two out of the three studies included in the analysis compared data on individuals with T1D from the Swedish Childhood Diabetes Register (18) with the overall population data obtained from the Swedish Total Population Register, matched by year of birth and municipality of residence at the time of diagnosis. To obtain key information on demographic, educational and socioeconomic status, the Registers were also linked to the Multi-Generation Register (19) and the Longitudinal Integration Database for Health Insurance and Labor Market Studies (20). In addition to the Registers, Persson et al. (16) linked data from the Swedish Medical Birth Register (21), and Lovén et al. (15) from the Swedish Register of Education (22).

The data presented in the third paper (17) derived from a larger evaluation study on transitional care for young adults with chronic diseases that used a mixed-methods design (23). Bronner et al. focused specifically on the data from the Dutch version of the Pediatric Quality of Life Inventory for young adults (PedsQL-YA) (24) questionnaire administered to young adults with T1D, healthy controls, and subjects with other chronic diseases, the most common being asthma, psychiatric disorders, digestive disorders, gastrointestinal diseases and skin diseases (17).

The findings of the three studies included in the scoping review revealed significant differences in educational, income and employment, and HRQoL outcomes for individuals with T1D, compared to their peers without diabetes, with age- and sex-specific differences emerging across most domains.

### 3.2 Education

According to Lovén et al. (15), individuals with T1D exhibit distinct patterns of educational achievement compared to age- and residence-matched peers without diabetes. While upper secondary school attendance showed no notable differences, university attendance was significantly lower among individuals with diabetes, and this affected both men and women, 30% vs. 35% and 40% vs. 45.8%, respectively. However, when adjusting for upper secondary grades, the estimates of lower university attendance for individuals with T1D, compared to controls, decreased by approximately one-third. Though higher grades were positively linked to university attendance, the relationship between grades and the chosen field of education was weaker. Interestingly, women with T1D were more likely to pursue health-oriented education, both at upper secondary and university levels, compared to both their peers without diabetes and men with T1D. Similarly, Persson et al. report that, by age 32, compared to age- and residence-matched controls without diabetes, both men and women with T1D had completed fewer years of schooling (mean 12.92 vs. 13.18, *p* < 0.001 for women and mean 12.45 vs. 12.63 *p* = 0.009 for men) and were less likely to hold a university degree (46% vs. 51%, *p* = 0.001 for women and 33% vs. 36% *p* = 0.016 for men) (16).

### 3.3 Employment

The educational challenges faced by individuals with T1D can have downstream effects on employment and income. Persson and colleagues observed that women with T1D were less likely to be employed and earned less than women without diabetes, particularly in later young adulthood (ages 27–32) (OR for employment 0.56; 95% CI 0.45–0.71; and 9.1%; 95% CI: 4.4–13.8% lower earnings). The presence of T1D did not affect employment in the male population, indeed, in the early young adult group (ages 19–26), the authors found a positive association between T1D and employment/earnings (OR for employment 1.19; 95% CI 1.06–1.34; and 3.0%; 95% CI: 0.2–6.0% higher earnings). This trend, however, reversed after the age of 27 when, even within the same area of occupation (categorized according to the Swedish standard classification of occupations) and adjusting for educational levels, individuals with T1D tended to have lower predicted income compared to peers without diabetes. This negative effect increases with disease duration, with 5.8% lower earnings after 15 years from diagnosis and 12.4% lower earnings after 25 years (16).

### 3.4 Health-related quality of life

The challenges faced by individuals with T1D extend beyond education, employment and income into their overall QoL. Bronner et al. assessed HRQoL using the Dutch version of the PedsQL-YA (24), a scale that consists of 23 items divided into four subscales: “physical health”, “emotional functioning”, “social functioning”, and “school/work functioning”. The multivariate regression analyses revealed that young adults with T1D obtained significantly different HRQoL scores, if compared to healthy individuals and to individuals with other chronic diseases. In particular: (i) individuals with T1D scored lower on overall HRQoL measures compared to healthy peers (*β* = −6.29; 95% CI: −8.74 to −3.85), but higher than individuals with other chronic diseases, although this difference had no clinical relevance; (ii) young adults with T1D scored worse than the healthy control group across all HRQoL domains except “social functioning” (*β* = −1.94; 95% CI: −4.72 to 0.84), the “school/work functioning” domain being the one with the most significant gap (*β* = −12.74; 95% CI: −15.82 to −9.66); (iii) while “emotional functioning” and “school/work functioning” scores of individuals with T1D were comparable to those of individuals with chronic diseases, “physical health” and “social functioning” scores were better; (iv) better overall HRQoL scores were observed in younger participants (*β* = 0.53; 95% CI: 0.04–1.03) and males (*β* = 4.07; 95% CI: 1.88–6.26). The challenges most frequently reported as experienced “almost always” and “often” by young adults with diabetes concerned the PedsQL-YA items “I have low energy” (26.1%), “I forget things” (17%) and “It is hard to pay attention at work or study” (13.3%). With regards to these items, significant differences were found between the T1D group and the healthy control group, whereas no significant differences emerged from the comparison with the chronic disease group (17).

## 4 Discussion

This scoping review aimed at examining the international literature on the long-term impacts of juvenile-onset T1D on education, employment, perceived QoL and psychosocial issues, with a special focus on potential barriers that individuals with T1D diagnosed in childhood or adolescence may face in adulthood. The article selection process revealed that a significant number of papers either did not identify gaps or assess any of the dimensions of interest in the relevant population. Other studies did not fulfill the criteria of including individuals with T1D aged 18 and over, or distinguishing between individuals with juvenile-onset T1D and individuals who had been diagnosed later in life, or considering adults with T1D as separate from individuals with type 2 diabetes in the results. Of the three studies included in the scoping review, two were based on data in the Swedish Childhood Diabetes Register on individuals born in the period 1962–1978, who would have been young adults between the early 80s and the late 90s. The third study, a cross-sectional study on HRQoL, derived its data from a larger mixed design evaluation study on transitional care.

Overall, the results of the articles selected for the analysis highlighted the impact of T1D on education, employment and perceived QoL in adults with juvenile-onset T1D, compared to individuals without diabetes.

With regards to formal education attainments, the study by Lovén et al. (15) reports lower university attendance in individuals with juvenile-onset T1D diagnosed during the years 1977–1990, compared to matched population controls. Similarly, Wennick et al. (25) associate the presence of childhood diabetes with a 7% lower probability of attaining the highest level of education and with a 7% higher probability of attaining the lowest level, compared to the general population within the same age group. Recently published data from the Italian National PASSI Survey for the years 2010–2018 and 2023 indicate that the percentage of juvenile-onset T1D adults with a university degree is lower, albeit not significantly (*p* = 0.06), than that of age and sex-matched individuals without diabetes (17% vs. 21%) (26). Counter to the above findings, a population-based study by Nielsen et al. (27) reports that adults with T1D are better educated compared to the general population, even after adjusting for sex and age, although the differences are relatively small and not significant. These contrasting results could reflect a progressive reduction in the academic achievements gap between individuals with T1D and the general population, perhaps due to improvements in self-management derived from advancements in glucose monitoring (28) and insulin delivery technologies (29), over the past two decades.

The impact of a diagnosis of T1D received during childhood or adolescence acquires additional levels of complexity during the transition into adulthood when individuals need to reconcile the demands of self-management and adherence to treatment guidelines with daily life, as the responsibility for self-management shifts fully from the legal guardian to the emerging adult (30). Daily insulin injections, glucose level checks, experiencing hypoglycemia may contribute to the feelings of isolation and disease-related stigma experienced by individuals with T1D during adolescence (31) that can carry over into adulthood (32). Despite its positive impact on self-management (29), even the use of wearable devices, such as the insulin pump, can carry a negative psychosocial burden (31). All of these aspects may negatively affect adherence to treatments and worsen HRQoL perception, with a severe impact on academic and working activities (33).

Access to the labor market is also critical for individuals with T1D who are less likely to be employed compared to the general population. This difference is dependent on sex and age and affects, in special measure, women and individuals aged ≥50 with T1D (27). According to Persson and colleagues, individuals with T1D also have lower predicted long-term income compared to peers without diabetes. This is especially true for women with T1D in later young adulthood (ages 27–32). On the contrary, an initial advantage is observed among males with T1D in early young adulthood (ages 19–26) in terms of employment and earnings. The advantage, however, tends to reverse after the age of 27 (16), suggesting shorter educational paths for these individuals, leading to earlier workforce entry and income generation in potentially less qualified positions, while non-diabetic peers are still in education. The social disadvantage deriving from lower employment, work profile and predicted income is further compounded by the burden of managing the disease and workplace demands. Hansen et al. (34) report lower lifetime income in adults with T1D, as well as increased absenteeism and presenteeism, and disability retirement, in the context of work life. Consistent with these findings, Brod and colleagues, when analyzing lost work time, missed meetings and unmet deadlines due to non-severe hypoglycemic events (NSHE), report loss in productivity in the range of $15.26 to $93.47 USD per NSHE event (35).

Evidence from qualitative studies highlights contextual factors influencing self-management practices and illness perception of people with T1D in work life (36–38). Young adults with T1D can experience difficulties with self-management routines and time tables in the workplace, mainly as a result of work pressure, the non-routine or physically demanding nature of some types of jobs and the work environment (37, 38) It has been suggested that the difficulty of reconciling work and self-management is a significant potential source of diabetes distress (DD). DD and work-related DD can act as separate intermediate pathways leading to intentional hyperglycemia at work and low glycemic control (39), confirming that work-related factors influence self-management among working people with T1D (36, 38). According to Sturt and colleagues, elevated DD is experienced by 20%–30% of people with T1D, with longer duration of diabetes, severe hypoglycemia, younger age and being female as the main risk factors for elevated DD in T1D individuals (40). A recent cross-sectional study on Hungarian patients with T1D by Losonczi and colleagues found a significant negative association between metabolic control and QoL and DD, using both physiological and behavioral indicators (41).

In their paper, Bronner et al. (17) specifically explore aspects related to HRQoL in young adults (19–28 years of age) with T1D. Compared to healthy controls, the T1D group scores worse across all HRQoL domains, especially in the “school/work functioning”, with the most frequently reported challenges being “forgetfulness” and “difficulty focusing at work or while studying”, in addition to one item from the Physical scale, namely “low energy”. These findings may find an additional explanation in the results of a study by Ferguson and colleagues, which detects a certain degree of cognitive impairment affecting T1D adults that may make it particularly challenging to work or study (42).

The degree to which physical and psychological well-being influence each other in the context of T1D is also the focus of other cross-sectional and qualitative studies on perceived health functioning (PHF) and HRQoL. The presence of childhood diabetes has been found to be associated with a 14% lower probability of a highest level assessment of own health later in life, although higher education seems to be associated with a 3% higher probability that the respondents will assess their health at the highest level (25). Hart et al. (43) report individuals with T1D having slightly poorer PHF with scores decreasing more rapidly over time, compared to the general population, especially with regards to the physical components, but not for the mental components. When controlling for sex and age, males and younger adults display better overall HRQoL scores, in particular in the “physical health” and “emotional functioning” sub-scales.

These results lend further support to previous evidence that HRQoL scores worsen with longer duration of T1D and that women with T1D, regardless of age, suffer worse physical and emotional burden than males with T1D (27). Age and being female are also identified by Joensen et al. (44) as factors affecting emotional burden in the T1D adult population, together with the presence of other chronic illnesses, low social support, low quality of life, low diabetes empowerment and high HbA1. Interestingly, age is negatively associated with emotional disfunction which is in line with the findings of other authors who report adolescents and younger adults feeling scared when thinking about living with diabetes and self-management, discouraged with their diabetes routine, and uncomfortable with diabetes-related interactions with family/friends (40, 45). The emotional burden of T1D has been investigated by some authors through the lens of stigma. According to Holmes-Truscott et al. (46), 62%–80% of adults with T1D report having “identity concerns” due to experiencing judgements when their diabetes becomes visible to others (e.g., when injecting insulin, checking glucose levels, experiencing hypoglycemia). Such experiences may lead individuals to hide or delay their diabetes self-management or avoid activities that would expose their condition. This is consistent with the findings of Hakkarainen and colleagues that report wage earners with T1D concealing their condition during their working career from colleagues and line managers, in particular in the youngest age group of 18–24 years (47).

## 5 Conclusion

This scoping review of available evidence on the impact of juvenile-onset T1D on adult life has found several gaps in the literature, highlighting the need for population analyses on up-to-date registry data and longitudinal studies designed specifically to explore the interplay between gaps and barriers and psychosocial outcomes in adults with T1D over time, complementing available qualitative studies. While it is a matter of debate whether individuals diagnosed with juvenile-onset T1D are at risk of lower educational achievements, the evidence supports the observation that they are more likely to experience reduced employment rates and lower income and lower overall QoL, compared to peers without diabetes. These gaps and barriers contribute to the difficulties that individuals with juvenile-onset T1D face as they grow into adulthood. Addressing gaps and barriers through structured transition programs from pediatric to adult care and multi-faceted interventions to foster more supportive and inclusive social and workplace environment may help improve occupational and psychosocial outcomes for this population (38).

Furthermore, as it seems that DD is present across the lifespan of individuals with T1D, this aspect should also be addressed, for example through HRQoL monitoring (48), in the early stages of diabetes and during the early years of adulthood in order to prevent the long-term negative effects of diabetes-related distress on self-management and HRQoL.

## Data Availability

The original contributions presented in the study are included in the article/Supplementary material, further inquiries can be directed to the corresponding authors.
